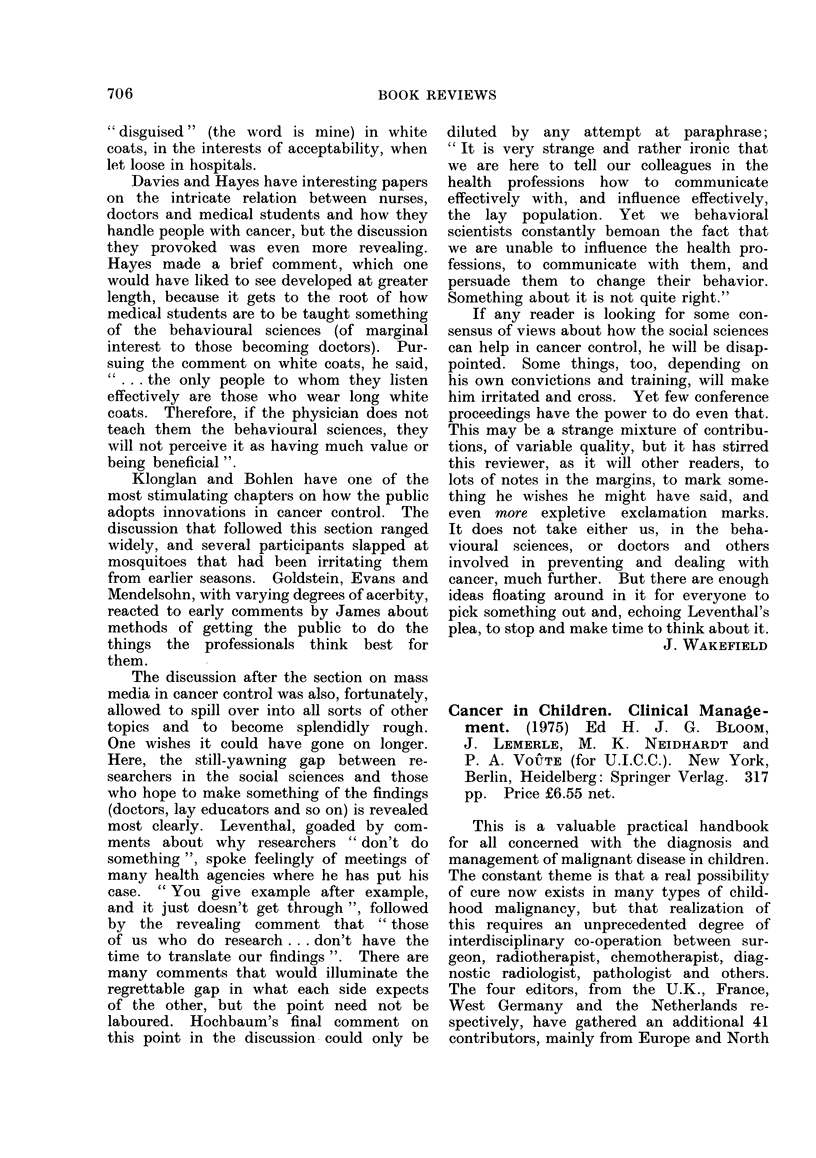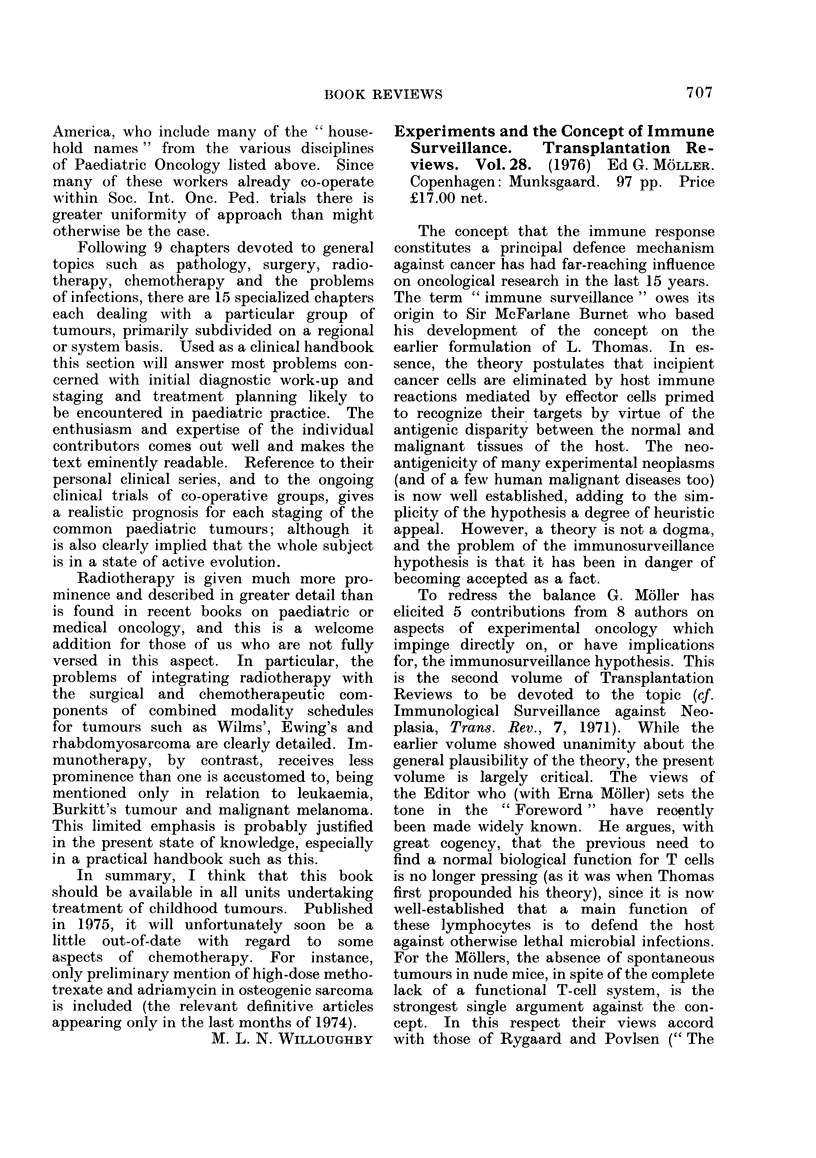# Cancer in Children. Clinical Management

**Published:** 1977-05

**Authors:** M. L. N. Willoughby


**Cancer in Children. Clinical Management.** (1975) Ed H. J. G. BLOOM, J. LEMERLE, M. K. NEIDHARDT and P. A. VOÛTE (for U.I.C.C.). New York, Berlin, Heidelberg: Springer Verlag. 317 pp. Price £6.55 net.

This is a valuable practical handbook for all concerned with the diagnosis and management of malignant disease in children. The constant theme is that a real possibility of cure now exists in many types of childhood malignancy, but that realization of this requires an unprecedented degree of interdisciplinary co-operation between surgeon, radiotherapist, chemotherapist, diagnostic radiologist, pathologist and others. The four editors, from the U.K., France, West Germany and the Netherlands respectively, have gathered an additional 41 contributors, mainly from Europe and North

America, who include many of the "household names" from the various disciplines of Paediatric Oncology listed above. Since many of these workers already co-operate within Soc. Int. Onc. Ped. trials there is greater uniformity of approach than might otherwise be the case.

Following 9 chapters devoted to general topics such as pathology, surgery, radiotherapy, chemotherapy and the problems of infections, there are 15 specialized chapters each dealing with a particular group of tumours, primarily subdivided on a regional or system basis. Used as a clinical handbook this section will answer most problems concerned with initial diagnostic work-up and staging and treatment planning likely to be encountered in paediatric practice. The enthusiasm and expertise of the individual contributors comes out well and makes the text eminently readable. Reference to their personal clinical series, and to the ongoing clinical trials of co-operative groups, gives a realistic prognosis for each staging of the common paediatric tumours; although it is also clearly implied that the whole subject is in a state of active evolution.

Radiotherapy is given much more prominence and described in greater detail than is found in recent books on paediatric or medical oncology, and this is a welcome addition for those of us who are not fully versed in this aspect. In particular, the problems of integrating radiotherapy with the surgical and chemotherapeutic components of combined modality schedules for tumours such as Wilms', Ewing's and rhabdomyosarcoma are clearly detailed. Immunotherapy, by contrast, receives less prominence than one is accustomed to, being mentioned only in relation to leukaemia, Burkitt's tumour and malignant melanoma. This limited emphasis is probably justified in the present state of knowledge, especially in a practical handbook such as this.

In summary, I think that this book should be available in all units undertaking treatment of childhood tumours. Published in 1975, it will unfortunately soon be a little out-of-date with regard to some aspects of chemotherapy. For instance, only preliminary mention of high-dose methotrexate and adriamycin in osteogenic sarcoma is included (the relevant definitive articles appearing only in the last months of 1974).

M. L. N. Willoughby